# Seizures in Alzheimer’s disease: is there more beneath the surface?

**DOI:** 10.1007/s00415-017-8694-6

**Published:** 2017-12-04

**Authors:** Marc Edwards, Neil P. Robertson

**Affiliations:** Institute of Psychological Medicine and Clinical Neurosciences, Cardiff University, Cardiff, CF14 4XW UK

In this month’s journal club we explore the significance of seizures in patients with dementia, events that can be distressing for both patients and relatives. Unprovoked seizures in Alzheimer’s disease are thought to affect up to a fifth of patients. However, seizures may be more difficult to identify so that rather than classical motor manifestations, patients may exhibit episodic confusion, behavioural change, increased drowsiness and/or clumsiness. The post-ictal state can also manifest as prolonged episodes of altered mental state which may further obscure or delay the diagnostic process leading to misdiagnoses with psychiatric disorders, metabolic disorders or transient ischaemic attacks. In addition, the effects of recurrent seizure activity on cognitive decline in these patients remain unclear. The first paper discussed looks at a novel way to investigate patients with Alzheimer’s disease and suspected seizure activity. In the second paper, Vossel et al. explore the impact of subclinical epileptiform activity and its effects on cognition. Finally, the third paper discussed investigates the pathological mechanism of cognitive impairment in temporal lobe epilepsy specifically considering whether the presence of a tauopathy in temporal lobe resections links epilepsy to the pathology underlying Alzheimer’s disease and chronic traumatic encephalopathy.

## Silent hippocampal seizures and spikes identified by foramen ovale electrodes in Alzheimer’s disease

Hypometabolism and cellular neurodegeneration in the temporal lobe are common features of both Alzheimer’s disease and epilepsy. Lam et al. hypothesise that intermittent temporal lobe dysrhythmia could account for the early fluctuations in cognition in patients with Alzheimer’s disease. In this paper, the authors report two cases of Alzheimer’s disease in which there was a high index of suspicion for occult seizure activity with no history of clinical seizures or epilepsy. Both patients were initially investigated with video EEG, which revealed infrequent epileptiform activity that was more prevalent during sleep. However, when bilateral foramen ovale electrodes were inserted, more frequent spike-and-wave activity was recorded arising from the mesial temporal lobe. In the first case, episodic confusion had led to a suspicion of subclinical seizure activity without scalp EEG evidence. With foramen ovale electrode placement, there was evidence of epileptiform activity whilst the patient was sleeping not present on the simultaneous scalp EEG. Levetiracetam was then introduced with the foramen ovale electrodes still in place subsequently revealing greatly reduced epileptiform activity and substantial clinical improvement. Similarly, foramen ovale insertion in the second patient resulted in the identification of a higher degree of subclinical seizure activity compared to scalp EEG. However, the second patient was unable to tolerate anti-epileptic therapy.

### Comment

The authors present a novel way to successfully determine whether patients with Alzheimer’s disease are having subclinical seizures where standard EEG has been insufficient for diagnosis. The bilateral foramen ovale electrodes are described as minimally invasive, however, they do require general anaesthesia for insertion as well as prophylactic antibiotics whilst in situ. The study is clearly limited by its small sample size and a larger sample size would be needed to validate the findings, but it may be difficult to identify patients as a result of potential ethical problems. In contrast, it could be argued that in the context of a substantial suspicion of seizure activity, a trial of anti-epileptic medication would be warranted and the first patient’s response to Levetiracetam was clearly encouraging. There is currently no single anti-epileptic drug of choice for seizures in Alzheimer’s disease which would also require large-scale studies to support an effective, evidence-based therapy.

*Lam AD* et al*. (2017) Nature Medicine. 23 (6): 678*–*680.*

## Incidence and impact of subclinical epileptiform activity in Alzheimer’s disease

Seizure activity is more common in dementia than in the general population and is associated with greater clinical decline. Understandably concern is raised when patients demonstrate clinically identifiable seizures but the impact of subclinical seizures is less clear. Vossel et al. hypothesise that subclinical epileptiform activity is more prevalent in Alzheimer’s disease than healthy controls and is associated with more rapid cognitive decline. As a standard 30-min scalp electroencephalography may be insufficient to detect subclinical seizures, participants underwent extended video EEG followed by magnetoencephalography (MEG). 33 individuals with Alzheimer’s disease were recruited. 42% had evidence of subclinical epileptiform activity compared to 10.5% of the 19 age-matched controls. Epileptiform activity was most often detected from the temporal lobe and more frequently seen during deeper stages of sleep. No subclinical seizures were recorded during the monitoring. The participants with Alzheimer’s disease and evidence of epileptiform activity tended to be younger but the link was not significant. On longitudinal evaluation, those individuals with subclinical epileptiform activity were significantly associated with an increased cognitive decline over 5 years measured with serial mini-mental state examination (MMSE) assessments.

### Comment

This study reveals a significant proportion of patients with Alzheimer’s disease that demonstrate subclinical epileptiform activity on extended monitoring. This is in keeping with the hypothesis that subclinical seizure activity in Alzheimer’s disease is currently under-estimated. The clinical significance of these findings is highlighted by the longitudinal data which shows an association between epileptiform activity and cognitive decline. However, there is not enough evidence from this study to establish a causal relationship. It could be argued that subclinical epileptiform activity may be a result of a more aggressive disease course. Further analysis of the relationship between seizure activity and cognitive decline in Alzheimer’s disease is required. As expected, extended EEG-monitoring and the combination of MEG and EEG was more sensitive in detecting subclinical epileptiform activity than standard scalp EEG. However, the relevance of this in a clinical setting depends on whether anti-epileptic medication is of benefit to these patients—an area which certainly warrants further exploration.

*Vossel KA* et al*. (2016) Annals of Neurology. 80: 858*–*870.*

## Hyperphosphorylated tau in patients with refractory epilepsy correlates with cognitive decline: a study of temporal lobe resections

The pathological features of Alzheimer’s disease, including amyloid plaques and neurofibrillary tangles consisting of hyperphosphorylated tau, are well described. The pathology underlying the cognitive decline seen in temporal lobe epilepsy (TLE) is less well understood. In a clinicopathological study, the authors of this paper investigate the relationship between TLE and tau pathology. The tissue from temporal lobe resections performed in patients with chronic refractory TLE was examined to assess the presence and extent of tau pathology using a modified tau score. Post-operative cognitive scores were then compared to the extent of tau pathology. The patients with higher tau scores were significantly associated with greater post-operative decline in verbal learning and recall. There was also a significant association between a history of secondary generalised seizures and a higher tau burden. With the majority of samples showing hippocampal sparing as well as an absence of beta-amyloid plaques, the pathology was not thought to be typical of that seen in Alzheimer’s disease. It was also not consistent with the classical findings of chronic traumatic encephalopathy. The authors suggest an epilepsy-specific tauopathy could underlie the cognitive decline in TLE and state that this warrants further investigation.

### Comment

This study offers an interesting insight into the pathology of patients with TLE with further evaluation of their cognitive function post-resection, setting this study apart from previous post-mortem studies. The tau burden observed was not significantly higher than in a post-mortem study of the general population which raises the possibility that hyperphosphorylated tau may not be the only factor contributing to cognitive decline in epilepsy. There appears to be significant association between generalised seizures, extent of tau pathology and cognitive decline but it is difficult to be certain of the causative relationship linking these features from this study alone. Although there were differences seen in terms of the pattern of pathology when compared to the primary age-related tauopathies, the study supports the idea that epilepsy has an integral neurodegenerative element. These findings and related studies will be of benefit to future therapies that target neurodegenerative changes and tau pathology.

*Tai XY* et al*. (2016) Brain. 139: 2441*–*2455.*

